# The cross-talk between methylation and phosphorylation in lymphoid-specific helicase drives cancer stem-like properties

**DOI:** 10.1038/s41392-020-00249-w

**Published:** 2020-09-30

**Authors:** Na Liu, Rui Yang, Ying Shi, Ling Chen, Yating Liu, Zuli Wang, Shouping Liu, Lianlian Ouyang, Haiyan Wang, Weiwei Lai, Chao Mao, Min Wang, Yan Cheng, Shuang Liu, Xiang Wang, Hu Zhou, Ya Cao, Desheng Xiao, Yongguang Tao

**Affiliations:** 1 Department of Pathology, Key Laboratory of Carcinogenesis and Cancer Invasion (Ministry of Education), Xiangya Hospital; Central South University, 410078 Hunan, China; 2 NHC Key Laboratory of Carcinogenesis (Central South University), Cancer Research Institute and School of Basic Medicine, Central South University, 410078 Changsha, Hunan China; 3 Postdoctoral Research Workstation, Department of Neurosurgery, Xiangya Hospital, Central South University, 410078 Hunan, China; 4 Department of Oncology, Institute of Medical Sciences, National Clinical Research Center for Geriatric Disorders, Xiangya Hospital, Central South University, 410008 Changsha, Hunan China; 5 Xiangya School of Pharmaceutical Sciences, Central South University, 410078 Changsha, China; 6 Hunan Key Laboratory of Tumor Models and Individualized Medicine; Hunan Key Laboratory of Early Diagnosis and Precision Therapy in Lung Cancer, Department of Thoracic Surgery, Second Xiangya Hospital, Central South University, 410011 Changsha, China; 7 Shanghai Institute of Material Medical, Chinese Academy of Sciences (CAS), 555 Zuchongzhi Road, Zhangjiang Hi-Tech Park, 201203 Shanghai, China

**Keywords:** Cancer stem cells, Lung cancer

## Abstract

Posttranslational modifications (PTMs) of proteins, including chromatin modifiers, play crucial roles in the dynamic alteration of various protein properties and functions including stem-cell properties. However, the roles of Lymphoid-specific helicase (LSH), a DNA methylation modifier, in modulating stem-like properties in cancer are still not clearly clarified. Therefore, exploring PTMs modulation of LSH activity will be of great significance to further understand the function and activity of LSH. Here, we demonstrate that LSH is capable to undergo PTMs, including methylation and phosphorylation. The arginine methyltransferase PRMT5 can methylate LSH at R309 residue, meanwhile, LSH could as well be phosphorylated by MAPK1 kinase at S503 residue. We further show that the accumulation of phosphorylation of LSH at S503 site exhibits downregulation of LSH methylation at R309 residue, which eventually promoting stem-like properties in lung cancer. Whereas, phosphorylation-deficient LSH S503A mutant promotes the accumulation of LSH methylation at R309 residue and attenuates stem-like properties, indicating the critical roles of LSH PTMs in modulating stem-like properties. Thus, our study highlights the importance of the crosstalk between LSH PTMs in determining its activity and function in lung cancer stem-cell maintenance.

## Introduction

Lung cancer remains the most common cancer and the top cause of cancer-related mortality worldwide in 2018 when both sexes are combined, with 2.1 million newly diagnosed cases (11.6% of the total cancer cases) and an estimated 1.8 million deaths (a mortality rate of ~18.4%).^1^ Lung cancer has two major types according to histopathology: small-cell lung cancer (SCLC) and non-small-cell lung cancer (NSCLC), which includes adenocarcinoma (ADC), squamous cell carcinoma (SCC) and large cell carcinoma (LCC), accounting for more than 80% of all lung cancer cases.^2^ While there are improvements with chemotherapy agents and radiotherapy, the majority of patients after diagnosis still lack effective therapies and have poor survival. To discover critical elements and molecular pathways that could be targeted for valid therapy, it is extremely urgent to understand molecular mechanisms and identify key factors that drive lung cancer pathogenesis.

A large number of studies have shown that resistance to chemoradiotherapy in lung cancer cells could play a vitally important role in weakening the efficacy of chemoradiotherapy, eventually leading to recurrence after treatment and poor survival.^3,4^ Additionally, increasing emerging evidence has shown that there exists a small population of lung carcinoma cells, called lung cancer stem-like cells, that can survive radiotherapy and/or chemotherapy and potentially propagate the tumor.^5^ Furthermore, numerous studies indicate that lung cancer stem cells are responsible for tumor propagation, metastasis, resistance to conventional therapy and tumor recurrence.^6,7^ Therefore, understanding the molecular pathways and identifying the key factors underlying the stemness properties of lung cancer cells may contribute significantly to the development of novel and effective therapeutic targets. While a number of stem cell-specific markers have been identified in lung cancer stem cells, such as CD166 and aldehyde dehydrogenase (ALDH), as well as ATP binding cassette subfamily G member 2 (ABCG2),^8,9^ to date, valid therapy targeting the eradication of lung cancer stem cells remains a tough challenge.

The chromatin remodeling protein LSH (also known as HELLS, PASG, SMARCA6), belonging to the SNF2 family of chromatin remodeling ATPases, is involved in normal embryonic development, with LSH-deficient mice displaying either postnatal lethality^10^ or premature aging.^11^ Furthermore, genetic mutation of LSH is responsible for human disease ICF syndrome (immunodeficiency, centromeric instability, facial anomalies).^12^ Molecularly, LSH can function through interactions with various molecules, for example, LSH, together with DNA methyltransferases, can regulate DNA methylation and transcriptional silencing.^13^ Moreover, the CDC7A/HELLS complex might have a role in DNA repair, especially in DNA DSB repair, and either CDC7A or HELLS deficiency may cause a C-NHEJ defect at DNA damage sites.^14^ Additionally, our previous studies showed that LSH could drive cancer metabolism and progression via involvement in apoptosis and ferroptosis.^15–21^ Recent research revealed that LSH was essential for ∆Np63α (a main p63 isoform) boosting stem-like characteristics and tumorigenesis of skin cells.^22^ In addition, LSH could also regulate the expression of genes vital for maintaining glioma stem cells through interactions with E2F3 and MYC.^23^ Nevertheless, the detailed functions of LSH, especially the mechanisms underlying its modulation of either cancer progression or cancer stem cell properties, have not been thoroughly explored.

Accumulating evidence has shown that posttranslational modifications (PTMs) of proteins have been implicated in the dynamic alteration of various protein properties and functions, such as protein stability, localization conformation states and activities. Recently, more than hundreds of PTMs have been reported, and the most common PTMs, including phosphorylation, ubiquitination, methylation, acetylation, and SUMOylation, have been shown to play critical roles in regulating almost all cellular processes, such as DNA repair, RNA processing, cell cycle progression, as well as transcription control.^24,25^ A protein may undergo diverse types of PTMs, and there exists crosstalk between these different modifications,^26–28^ which makes PTMs particularly significant for many biological processes. Among the many potential mechanisms implicated in regulating the characteristics of cancer stem cells, posttranslational modifications of stem cell-associated molecules play significant roles, for example, SUMOylation of nestin contributes to the association between nestin and c-Myc by regulating its translocation, which ultimately modifies the stemness and paclitaxel sensitivity of brain cancer stem cells.^29^ Sox2, a known key stem cell-related factor, is capable of being both methylated and phosphorylated, and a switch between these two PTMs determines Sox2 stability and functions.^30^ Furthermore, AKT-catalyzed Sox2 phosphorylation antagonizes UBR5-mediated ubiquitination to stabilize Sox2 and thereby enhance cancer cell stemness.^31^ Moreover, OCT4, a vital stemness marker, can be ubiquitinated by CHIP, and low expression of CHIP increases OCT4 stability, thus promoting a stem cell phenotype in breast cancer.^32^

Protein arginine methylation has emerged as an important PTM, can be detected both on histone and nonhistone proteins and is involved in various biological processes, such as DNA repair, RNA processing, chromatin regulation and signal transduction. As reported, thus far, nine protein arginine methyltransferases (PRMTs) have been identified in human,^33^ whereas just one demethylase, namely, Jumonji domain containing protein 6 (JMJD6), was found to have potential arginine demethylation activity in vivo.^34^ In recent years, a compelling amount of data has indicated that protein arginine methylation as well as demethylation play vital roles in tumorigenesis.

Here, we reported that chromatin remodeling protein LSH could be methylated by PRMT5 and demonstrated that PRMT5 efficiently monomethylates LSH at the R309 residue. Additionally, LSH could also be phosphorylated by kinase MAPK1 at the S503 residue. Furthermore, S503 phosphorylation (pS503) can antagonize R309 methylation (R309me1) to influence LSH activity to sustain stem cell properties in lung cancer. Therefore, our study presents evidence of the crosstalk between LSH methylation and phosphorylation, and these two modifications may play significant roles in modifying LSH activity for lung cancer stem cell maintenance.

## Results

### LSH interacts with PRMT5 in vitro and in vivo

**Fig. 1:**
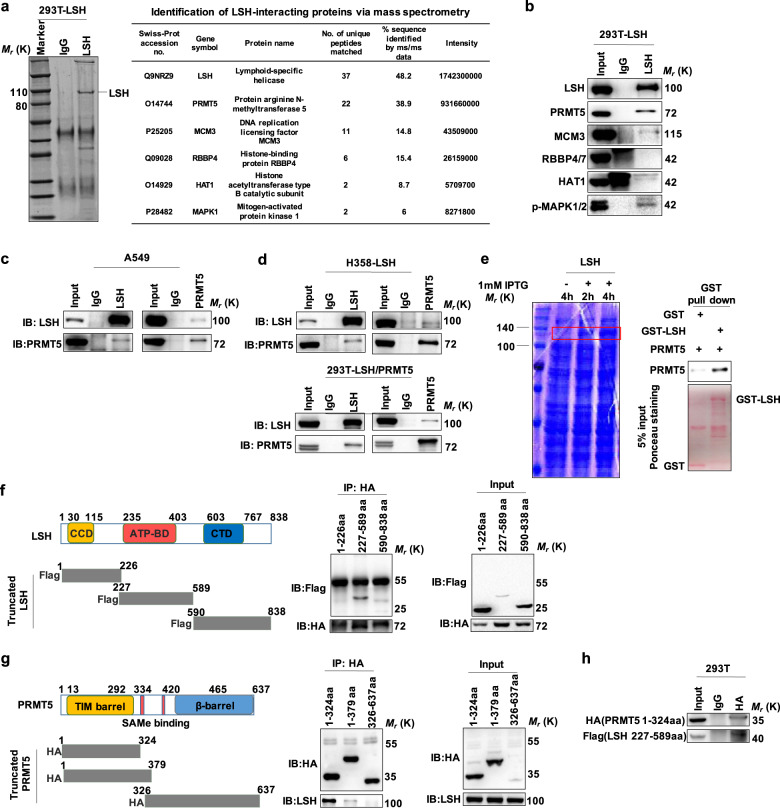
LSH interacts with PRMT5 in vitro and in vivo. **a** A mass spectrometry assay was performed, and the potentially interactive proteins with LSH were listed. **b** IP analysis for LSH and its interactive proteins in 293T cells ectopically expressing LSH, followed by IB with indicated antibodies. Immunoglobulin (IgG) was employed as an isotype control. **c**, **d** Co-IP and IB analysis of endogenous or exogenous LSH interaction with PRMT5. LSH or PRMT5 immunoprecipitated from various cells, including A549 (**c**), H358 and 293T cells (**d**), was immunoblotted with anti-PRMT5 or anti-LSH antibodies. **e** GST pull down assay of the direct interaction between full-length LSH and PRMT5. GST-LSH expressed in *E. coli* pulled down PRMT5 with HA tag expressed in 293T cells. **f** Schematic representation of Flag-tagged human LSH truncation derivatives and IP analysis followed by an IB assay for interaction between full-length PRMT5 and LSH fragments. LSH amino acids 227–589 are vital for its interaction with PRMT5. **g** Schematic representation of the HA-tagged human PRMT5 fragments and IP analysis followed by IB assay for interaction between full-length LSH and PRMT5 truncation mutants. PRMT5 amino acids 1–324 are necessary for its binding to LSH. **h** HA-tagged amino acids 1–324 of PRMT5 were immunoprecipitated from 293T, and LSH amino acids 227–589 were detected by probing with the anti-Flag antibody

To identify LSH-interacting proteins to better understand LSH activity and its potential mode of regulation, we performed immunoprecipitation (IP) experiments using an anti-LSH antibody and conducted a systematic mass spectrometry analysis in 293T cells stably expressing LSH. Notably, numerous molecules were found to coprecipitate with LSH, including PRMT5, minichromosome maintenance 3 (MCM3), RB binding protein 4/7 (RBBP4/7), histone acetyltransferase 1 (HAT1) and MAPK1 (Fig. 1a). Then, an immunoprecipitation assay using an anti-LSH antibody was performed to further confirm the proteins interacting with LSH. Eventually, we found that PRMT5 and MCM3 as well as MAPK1 could interact with LSH, and PRMT5 had a robust interaction with LSH (Fig. 1b). In addition, the interaction between PRMT5 and LSH was further verified by blotting endogenous and exogenous PRMT5/LSH precipitated with LSH/PRMT5-specific antibodies (Fig. 1c, d). Additionally, a GST pull down assay demonstrated that PRMT5 could be pulled down by LSH (Fig. 1e), suggesting there is direct binding between LSH and PRMT5 in vitro and in vivo. To specifically identify the interactive motif, we generated three Flag-tagged deletion mutants of LSH and several HA-tagged deletion mutants of PRMT5 and cotransfected these truncated mutants into 293T cells; through coimmunoprecipitation experiments, we finally found that the middle fragment (amino acids 227–589) of LSH and the N-terminus (amino acids 1–324) of PRMT5 are critical to modulate the interaction between LSH and PRMT5 (Fig. 1f–h). Collectively, these data suggest that LSH could directly interact with PRMT5.

### PRMT5 methylates LSH at arginine 309

**Fig. 2:**
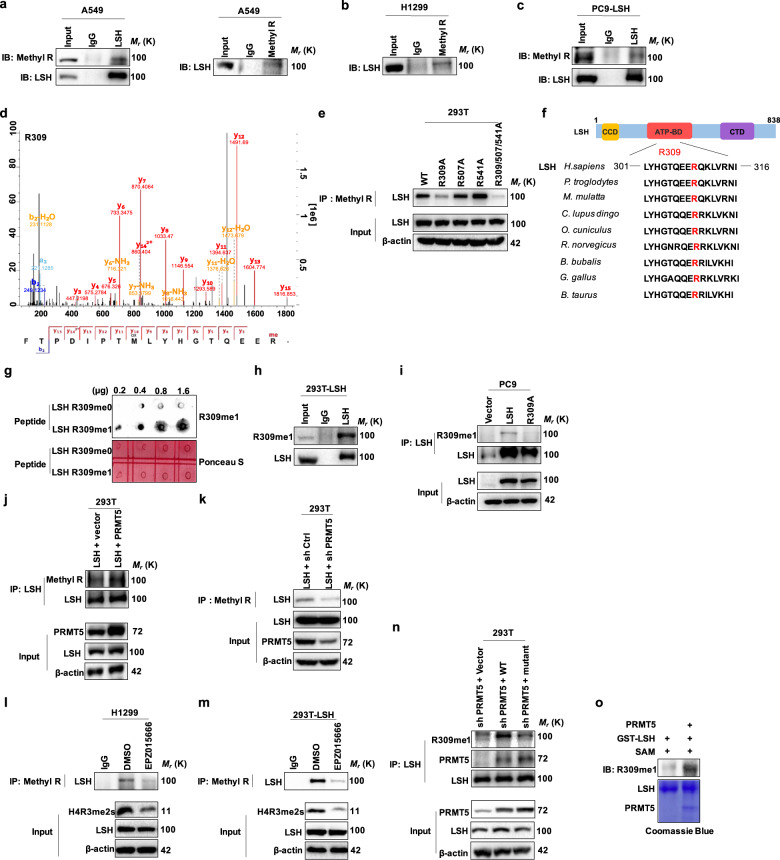
PRMT5 monomethylates LSH at R309. **a**–**c** Endogenous, exogenous LSH or arginine methylated proteins were immunoprecipitated from A549 (**a**), H1299 (**b**) and PC9 cells (**c**) by anti-LSH antibody or anti-pan mono methyl arginine antibody (Methyl R), and the arginine methylation status of LSH was examined with a Methyl R or LSH antibody. **d** Mass spectrometric analysis showing LSH R309 methylation in A549 cells. **e** IB analyses of immunoprecipitated proteins, obtained from 293T cells transiently transfected with plasmids expressing LSH WT, LSH mutants including R309A, R507A, R541A, and R309/507/541A. **f** Alignment of the consensus LSH amino acid sequences around the arginine 309 residue highlighted in red among various species. **g** The synthetic LSH peptides containing the R309 residue without or with methylation were used in dot blot analysis to validate the validity and specificity of the anti-LSH R309me1 antibody. **h**, **i** Proteins derived from 293T cells ectopically expressing LSH (**h**), and PC9 cells stably expressing LSH WT or LSH R309A (**i**) were subjected to IP analysis, followed by IB assay with the generated anti-LSH R309me1 antibody. **j**, **k** 293T cells ectopically expressing human PRMT5 (**j**) and shPRMT5 (**k**) were collected and subjected to an immunoprecipitation assay with the Methyl R antibody, followed by IB analysis with the anti-LSH antibody to identify the effect of PRMT5 on LSH arginine methylation. **l**, **m** Endogenous, exogenous arginine methylated proteins were immunoprecipitated from H1299 (**l**) and 293T cells (**m**) treated with EPZ015666 (specifically inhibit PRMT5 methyltransferase activity) by anti-pan monomethyl arginine antibody (Methyl R), and the arginine methylation status of LSH was examined with LSH antibody. **n** 293T cells ectopically expressing human PRMT5 WT and mutant after knocking down PRMT5 were collected and subjected to an immunoprecipitation assay to identify the effect of PRMT5 on LSH arginine methylation. **o** Purified GST-LSH, HA-PRMT5 fusion protein and SAM were subjected to in vitro methylation analysis with LSH R309me1 antibody to detect LSH methylation. Immunoblotting (top panel) and Coomassie Brilliant blue staining (bottom) were shown

Protein arginine methyltransferase 5 (PRMT5) is a member of the type II protein arginine methyltransferases, which can catalyze both ω-N^G^-monomethyl arginine (MMA) and symmetric ω-N^G^,N^G^-dimethylarginine (SDMA) to methylate a variety of substrates including histones and nonhistones.^35–37^ Therefore, we investigated whether LSH is a substrate of PRMT5. First, to identify whether LSH could be methylated at arginine sites in cells, we immunoprecipitated arginine methylated proteins and examined the presence of LSH using an LSH-specific antibody. In turn, we also immunoprecipitated LSH and detected its methylation status with a specific pan-arginine methylation antibody in A549 cells. The results showed that LSH could be methylated at arginine sites (Fig. 2a). Similarly, consistent results were obtained in H1299 cells and PC9 cells stably expressing LSH (Fig. 2b, c), indicating that both endogenous and exogenous LSH could be methylated at arginine sites.

Next, a mass spectrometry assay was employed to identify LSH methylation sites. Ultimately, some methylated sites, but only three monomethylated arginine sites in total, were identified, including R309, R507, and R541 (Fig. 2d and Supplementary Fig. S2a). To determine the importance of R309, R507, and R541 for LSH arginine methylation, LSH R309A, R507A, R541A, and R309/507/541A mutations were generated and ectopically expressed in 293T cells, which were applied in immunoprecipitation experiments to detect LSH methylation levels. Interestingly, compared with wild-type LSH (WT), the R309A and R309/507/541A mutants presented reduced methylation levels, while the R507A and R541A mutants exhibited no significant reductions in LSH methylation levels (Fig. 2e and Supplementary Fig. S2b), indicating the R309 residue of LSH is critical for LSH methylation. Moreover, the R309 site is highly conserved in eukaryotic organisms (Fig. 2f).

To further explore and elucidate the significance of LSH arginine methylation in cells, we generated a R309-specific mono-methylation antibody of LSH (anti-R309me1 antibody), which was verified by dot blot and immunoprecipitation assays. The dot blot assay showed that this antibody could specially recognize a synthetic peptide with R309 methylation (R309me1: TQEE (meR) QKLVRN), but not a nonmethylated peptide (R309me0: TQEERQKLVRN) (Fig. 2g). Additionally, an immunoprecipitation experiment also showed that this specific antibody could recognize LSH methylation in cells (Fig. 2h), and compared with WT, the R309A mutation displayed markedly decreased or even absent LSH methylation levels in PC9, 293T as well as H358 cells (Fig. 2i and Supplementary Fig. S2c, d).

Furthermore, to attempt to establish whether PRMT5 is responsible for LSH methylation in cells, we conducted an immunoprecipitation assay in 293T cells, in which PRMT5 was either knocked down or overexpressed. As expected, we observed that LSH methylation levels highly increased in 293T cells when PRMT5 was overexpressed (Fig. 2j), on the contrary, depletion of PRMT5 led to a strong reduction of LSH methylation levels (Fig. 2k). Furthermore, EPZ015666 targeting PRMT5 to specifically inhibit its methyltransferase activity was used to identify this function of PRMT5. Through co-immunoprecipitation experiments, we finally found that EPZ015666 could strongly decrease the level of LSH arginine methylation in A549, H1299 cell lines and 293T cells transiently transfected with LSH (Fig. 2l and m, Supplementary Fig. S2e). Consistently, the PRMT5 mutant (PRMT5 T139/144A), which the activity of methyltransferase was drastically decreased,^38^ showed an attenuated ability to methylate LSH (Fig. 2n). Furthermore, in vitro methylation analysis as well confirmed that PRMT5 could methylate LSH at R309 in vitro (Fig. 2o). Taken together, these findings indicate that PRMT5 could serve as a methyltransferase responsible for R309 methylation of LSH.

### LSH is phosphorylated at serine 503

**Fig. 3:**
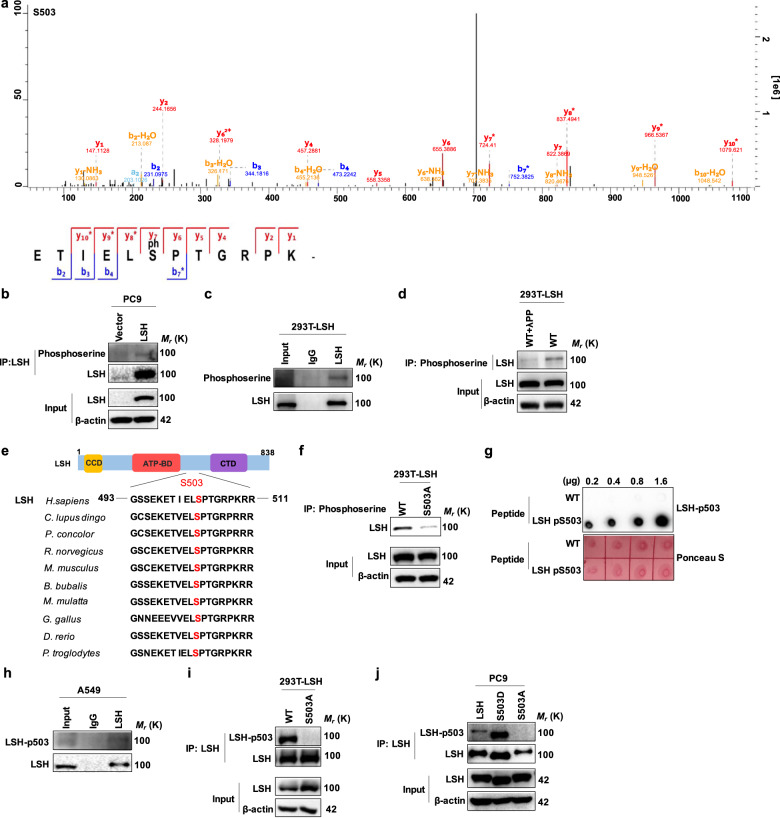
LSH is phosphorylated at serine 503. **a** Mass spectrometric analysis showing LSH S503 phosphorylation in A549 cells. **b**, **c** IB analysis of anti-LSH antibody immunoprecipitated proteins using anti-phosphoserine antibody in PC9 cells stably expressing LSH (**b**) and 293T cells transiently overexpressing LSH (**c**). **d** Lysates of 293T cells transiently transfected with LSH were treated with or without Lambda Protein Phosphatase (λPP) for the indicated time, and then IP and IB assays were performed to detect LSH phosphorylation. **e** Alignment of the consensus LSH amino acid sequences around the serine 503 residue highlighted in red in different species. **f** IB analyses of immunoprecipitated proteins, obtained from 293T cells transiently transfected with plasmids expressing LSH WT or LSH S503A mutant. **g** The synthetic LSH peptides bearing the S503 residue without or with phosphorylation were applied to dot blot analysis for validating the validity and specificity of anti-LSH-p503 antibody. **h** Endogenous LSH derived from A549, which possessed high LSH expression relative to other lung cancer cells, was immunoprecipitated by the anti-LSH antibody, and IB analysis was conducted with the generated anti-LSH-pS503 antibody. **i**, **j** 293T cells ectopically expressing LSH WT or LSH S309A (**i**), and PC9 cells stably expressing LSH WT, LSH S503D or LSH S503A (**j**), were subjected to IP analysis, followed by an IB assay with the generated phosphorylation antibody

Interestingly, phosphorylation of serine residue 503 (S503) of LSH was also detected in the results of a mass spectrometry assay aimed at identifying LSH methylation sites (Fig. 3a). To determine whether LSH could be phosphorylated, we immunoprecipitated LSH either transfected into 293T cells or stably expressed in PC9 cells in which endogenous LSH expression was relatively low, and we detected LSH phosphorylation status through an IB assay using a phosphoserine antibody. As expected, we observed that LSH could be phosphorylated in both PC9 and 293T cells (Fig. 3b, c). Meanwhile, LSH phosphorylation levels were reduced significantly after treatment with Lambda Protein Phosphatase (λPP) (Fig. 3d). In addition, S503 and its surrounding amino acid sequences exhibited remarkable conservation when LSH amino acid sequences from several eukaryotic organisms were compared, indicating potential evolutionary significance (Fig. 3e). To verify whether LSH could be phosphorylated at S503, we replaced the serine residue at position 503 with alanine (S503A) and performed an immunoprecipitation assay. Compared with WT, the phosphorylation level of the S503A mutant was markedly reduced (Fig. 3f).

To further confirm these findings, we generated a specific antibody that could recognize S503 phosphorylation of LSH (anti-LSH-pS503 antibody). Dot blot analysis demonstrated that this specific antibody could effectively recognize a synthetically phosphorylated peptide at S503 (KETIEL (pS) PTGR), but not a nonphosphorylated peptide (KETIELSPTGR) (Fig. 3g). Likewise, it could also detect endogenous LSH phosphorylation in A549 cells (Fig. 3h). Moreover, when LSH WT and the LSH S503A mutant were respectively expressed in 293T cells, this antibody identified phosphorylated WT, whereas it could not recognize the nonphosphorylated S503A mutant (Fig. 3i). We additionally substituted serine either with aspartic acid (S503D) or glutamic acid (S503E) at position 503 of LSH to make a phosphorylation mimetic mutant. However, only S503D mutant can successfully mimic LSH serine phosphorylation at S503 site. Consequently, we chose to apply S503D mutant to our experiments and observed that the phosphorylation level of the S503D mutant was sharply increased compared with that of the WT, while in contrast, the phosphorylation level of the S503A mutation was diminished or even absent in PC9 cells in which LSH WT or mutants was stably expressed (Fig. 3j). Collectively, these data demonstrate that LSH could be phosphorylated at serine residue 503.

### MAPK1 directly phosphorylates LSH at serine 503

**Fig. 4:**
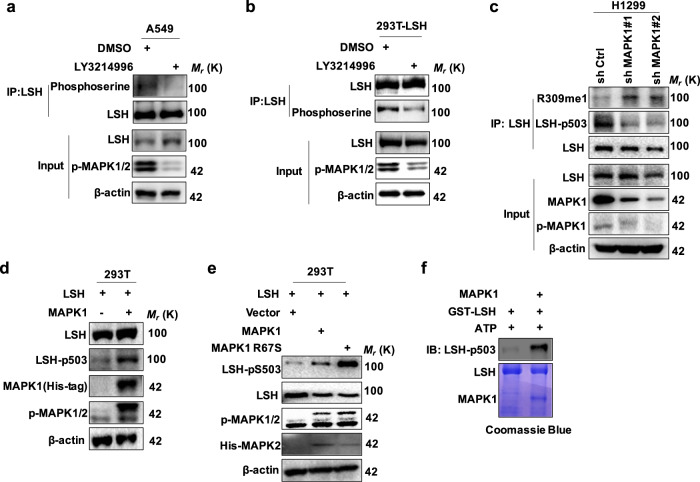
MAPK1 directly phosphorylated LSH at serine 503. **a**, **b** Endogenous and exogenous LSH were respectively immunoprecipitated from A549 (**a**) and 293T cells (**b**) after treatment with the MAPK1/2 inhibitor LY3214996 for the indicated time, and then IB assay was carried out to detect the phosphorylated LSH with anti-phosphoserine antibody. **c** IP and IB analysis of the methylated and phosphorylated LSH in H1299 cell lines, which MAPK1 was stably knocked down using MAPK1 short hairpin RNAs (shRNA). **d**, **e** IB analysis for LSH phosphorylation with the generated antibody LSH-pS503 in 293 T cells cotransfected with LSH and MAPK1 (**d**) or MAPK1 mutant (**e**). **f** Purified GST-LSH, His-MAPK1 fusion protein and ATP were subjected to in vitro kinase assay with LSH-pS503 antibody to detect LSH phosphorylation. Immunoblotting (top panel) and Coomassie Brilliant blue staining (bottom) were shown

To further investigate which kinase is responsible for phosphorylating LSH at S503, we carried out a computational prediction assay of phosphorylation sites with their cognate protein kinases (PKs). Notably, several kinases that may have a high potential to be implicated in this posttranslational modification were identified, including a component of inhibitor of nuclear factor kappa B kinase complex (IKKα), MAPK1 and cyclin dependent kinase 2/5 (CDK2/5) (Supplementary Fig. S3a). These protein kinase inhibitors therefore were applied to determine the major kinase that could phosphorylate LSH at serine residue 503. Interestingly, an immunoprecipitation assay was performed, and the results showed that neither roscovitine (a specific inhibitor of CDK5 and CDK2) nor IKKα inhibitor could impact LSH phosphorylation in 293T cells after treatment with roscovitine or IKKα inhibitor (Supplementary Fig. S3b–d). Nevertheless, after treatment with LY3214996 (an inhibitor of MAPK1/2), the LSH phosphorylation level was greatly decreased in A549 cells that just expressed endogenous LSH (Fig. 4a). Similar results were obtained in 293T cells transiently expressing LSH (Fig. 4b and Supplementary Fig. S3e).

To further investigate the role of MAPK1 in catalyzing LSH S503 phosphorylation, we stably knocked down MAPK1 using MAPK1 short hairpin RNAs (shRNA) in H1299 cell line to establish whether endogenous MAPK1 is involved in S503 phosphorylation. The results demonstrated that knocking down MAPK1 significantly decreased LSH S503 phosphorylation level (Fig. 4c and Supplementary Fig. S3f). Then both LSH and MAPK1 were cotransfected into 293T cells, IB analysis using the LSH-pS503 antibody was performed and the results as well demonstrated that MAPK1 overexpression could notably induce LSH S503 phosphorylation (Fig. 4d). Consistently, a point mutant of MAPK1 (MAPK1 R67S), which could augment MAPK1 autophosphorylation,^39^ dramatically increased the phosphorylation level of LSH (Fig. 4e). Furthermore, GST-LSH and His-MAPK1 fusion protein were applied to in vitro kinase assay, the results demonstrated that MAPK1 could phosphorylate LSH at S503 in vitro (Fig. 4f). Additionally, as shown in Fig. 1a, b, MAPK1 was found to interact with LSH. Together, these results coherently support the notion that MAPK1 may interact with LSH and serve as a major kinase contributing to the catalysis of LSH S503 phosphorylation.

### MAPK1-mediated LSH phosphorylation crosstalk with methylation of LSH by PRMT5

It is well established that there is a complex network between protein posttranslational modifications, and these PTMs are always synergistically or antagonistically involved in modulating protein activity.^40–42^ Recent studies revealed that AKT K140/142 methylation could crosstalk with AKT T308 phosphorylation to activate its oncogenic functions,^26^ and AKT K64 methylation plays vital roles in AKT T308 phosphorylation as well.^27^

**Fig. 5:**
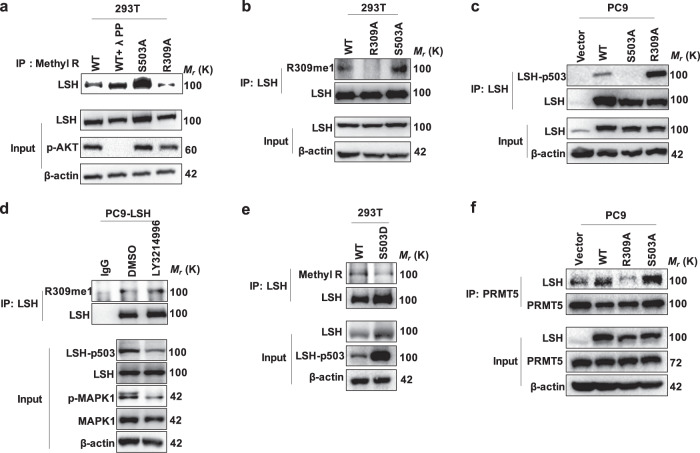
ERK2-mediated LSH phosphorylation crosstalk with methylation of LSH by PRMT5. **a** Methyl R antibody immunoprecipitated proteins were applied to IB analysis using an anti-LSH antibody to detect LSH methylation in 293T cells transfected with LSH mutants including R309A as well as S503A, or LSH WT, which were treated without or with λPP. **b** Anti-LSH antibody immunoprecipitated proteins obtained from 293T cells transiently transfected with plasmids expressing LSH WT, R309A or S503A were subjected to IB assay with the generated LSH R309me1 antibody. **c** Anti-LSH antibody immunoprecipitated proteins obtained from PC9 cells stably expressing Vector, LSH WT, R309A or S503A were subjected to IB assay with the generated anti-LSH-pS503 antibody. **d** IP followed by IB assay in PC9 cell lines stably expressing LSH after treatment with the MAPK1/2 inhibitor LY3214996 for the indicated time. **e** Immunoprecipitated proteins derived from 293T cells transfected with LSH WT or LSH S503D were used for IB analysis with the anti-LSH-pS503 antibody and anti-Methyl R antibody. **f** IP followed by IB assay of interaction between PRMT5 and LSH WT or LSH mutants in PC9 cells stably expressing Vector, LSH WT, R309A or S503A

In our study, we indicated that LSH could be methylated at R309 as well as phosphorylated at S503. Thus, we speculated that there may be a relationship between its methylation and phosphorylation. To determine whether LSH R309me1 could crosstalk with LSH-pS503, we performed an immunoprecipitation assay in 293T cells ectopically expressing LSH WT or mutants using the specific pan-arginine methylation antibody, followed by IB analysis. We observed that both the phosphorylation-deficient LSH mutant (S503A) and the WT treated with protein phosphatase λPP displayed dramatically elevated LSH methylation relative to the WT with no treatment, whereas the methylation-deficient LSH mutant (R309A) exhibited attenuated methylation of LSH (Fig. 5a and Supplementary Fig. S4a).

To further test this conclusion, we carried out the same experiment assay but applied the generated LSH-pS503 and LSH R309me1 antibodies. Of note, loss of LSH phosphorylation at S503 apparently facilitated LSH R309 methylation both in 293T and PC9 cells (Fig. 5b and Supplementary Fig. S4b) and weakened LSH methylation at R309 was accompanied by enhanced LSH phosphorylation at S503 in PC9 cells (Fig. 5c). Besides, both knocking down MAPK1 and inhibiting MAPK1 activity could elevate the level of LSH R309 methylation (Figs. 4c and 5d). Moreover, the exogenous phosphomimic LSH S503D mutant remarkably impaired LSH R309 methylation (Fig. 5e). These results along with the above findings suggest that LSH S503 phosphorylation may antagonize LSH R309 methylation. Furthermore, we demonstrated that the interaction between LSH and PRMT5 was weakened in the methylation-deficient LSH R309A mutant, while in contrast, the binding of LSH with PRMT5 was enhanced in the phosphorylation-deficient LSH S503A mutant (Fig. 5f). Therefore, we speculate that S503A mutant may affect R309me1 level through changing the interaction between PRMT5 and LSH. However, the specific mechanisms still need to be studied and verified further. Taken together, these data indicate that there exists crosstalk between LSH R309 methylation and LSH S503 phosphorylation, additionally, these two modified sites potentially play crucial roles in regulating the interaction between LSH and PRMT5.

### LSH promotes lung cancer stem-like properties through its crosstalk between methylation and phosphorylation

**Fig. 6:**
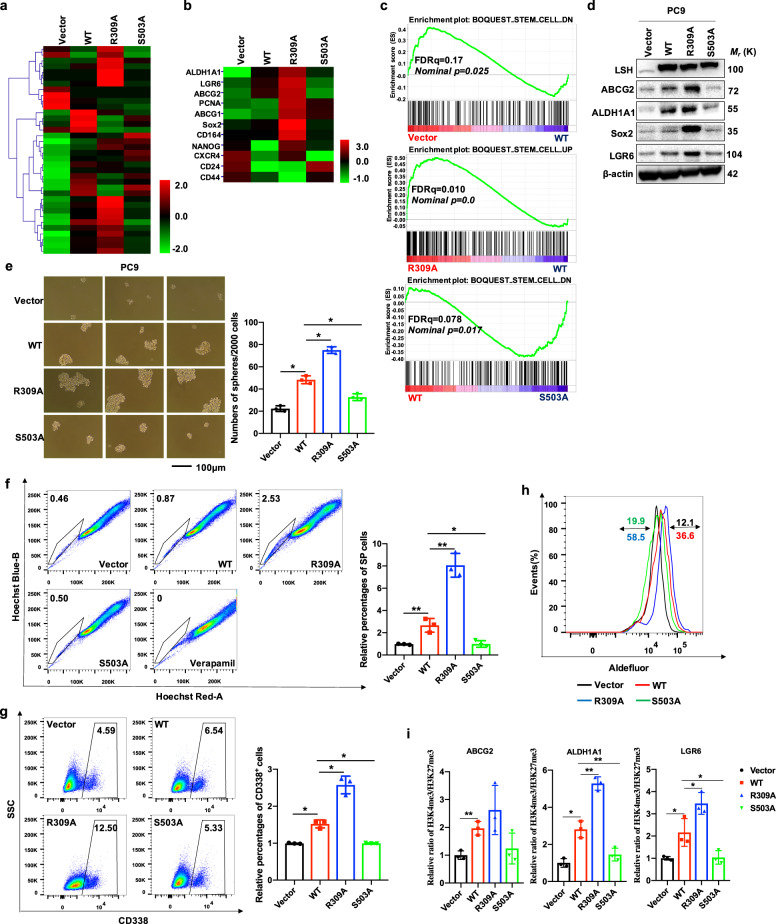
LSH methylation and phosphorylation modulates the lung cancer stem cell phenotype in vitro. **a**, **b** Heatmap of RNA-seq showing the differentially expressed genes (**a**) and stemness-related genes (**b**) in PC9 cells stably expressing Vector, LSH WT, R309A or S503A. **c** Gene Set Enrichment Analysis (GSEA) of RNA-seq data from PC9 cells as mentioned in **a** were performed using the gene sets of stem cell signatures. **d** The protein levels of stemness-related genes as indicated were examined by IB analysis in PC9 cells as mentioned in **a**. **e** Representative images of the tumor sphere formation assay in PC9 cells expressing LSH WT or mutants as mentioned in **a** were taken (left), and the numbers of formed spheres were counted and summarized in the bar graph (right, *n* = 3). **f** Representative images of flow cytometry analysis for the sorted SP population identified by Verapamil gating are shown, in which the population of SP cells was significantly reduced (left). The results were summed up in the bar graph. (right, *n* = 3). **g** Representative figures of the flow cytometry assay for the detection of CD338 positive cells in PC9 cells mentioned in **a** are displayed (left), and the results were quantified in the bar graph (right). **h** Representative Aldefluor assay to determine ALDH activity was conducted in PC9 cells stably expressing Vector, LSH WT or LSH mutants. **i** ChIP analysis in PC9 cells stably expressing Vector, LSH WT or LSH mutants was performed to detect H3K27me3 and H3K4me3 binding to stemness-related genes as indicated

To further assess the functional significance of LSH S503 phosphorylation and R309 methylation, we performed gene expression profiling analysis of PC9 cells stably expressing Vector, LSH WT, LSH R309A, and LSH S503A, followed by Gene Ontology (GO) enrichment analysis as well as transcriptome and gene set enrichment analysis (GSEA). RNA sequencing assay demonstrated that stem cell-associated factors notably changed, including ABCG2, SRY-box transcription factor 2 (SOX2), aldehyde dehydrogenase 1 family member A1 (ALDH1A), as well as leucine rich repeat containing G protein-coupled receptor 6 (LGR6) (Fig. 6a, b). Moreover, GO analysis revealed that LSH and mutants alike were implicated in modulating stem cell maintenance (Supplementary Fig. S5a), and GSEA analysis also demonstrated that LSH WT and mutants were capable of modulating the expression of genes enriched and involved in maintaining stem cell properties (Fig. 6c). These results indicated that compared with vector control, ectopic expression of LSH WT and the LSH R309A mutant could induce transcription of these genes; the LSH R309A mutant had a greater capability than LSH WT to promote the activation of stem cell markers, whereas the LSH S503A mutant may lead to downregulation of these genes. Consistent with these results, IB analysis and RT-qPCR revealed that both protein and mRNA levels of these factors associated with stemness were upregulated in the LSH R309A mutant but downregulated in the LSH S503A mutant relative to LSH WT (Fig. 6d and Supplementary Fig. S5b). These data indicate that LSH may be involved in modulating lung cancer stemness, and LSH R309 methylation suppresses this biological function, while LSH S503 phosphorylation enhances the cancer stem cell phenotype.

To exclude the possibilities that the point mutation of LSH may lead to the change of LSH activity, the localization of LSH in PC9 cell lines stably expressing LSH WT and mutants was detected. Subcellular location study indicated that both LSH WT and mutants were mostly distributed in the nucleus (Supplementary Fig. S5c), which means LSH mutants may do not influence its localization. Additionally, in our preliminary work we found that LSH overexpression significantly increased the expression levels of fatty acid desaturase 2 (FADS2) and FADS5 (also known as sterol-CoA desaturase 1, SCD1).^18^ RT-qPCR analysis was added in our experiments to explore whether LSH mutants could also be able to affects SCD1 and FADS2 expressions at the transcriptional level, and the results demonstrated that both LSH WT and mutants could be able to affects ferroptosis related genes expressions (Supplementary Fig. S5d). All these data partly supported that LSH mutants may not generate PTM-independent effect.

We subsequently verified the biological function of LSH and its two posttranslational modifications in lung cancer stemness. First, we carried out a tumor sphere forming assay and found that PC9 cells ectopically expressing WT displayed greater capability of forming tumor spheres relative to empty vector control; moreover, the methylation-deficient R309A mutant greatly augmented the LSH-promoted sphere-forming frequency, whereas the phosphorylation-deficient S503A mutant significantly attenuated LSH-facilitated sphere formation ability (Fig. 6e). Previous studies have shown that side population (SP) can be applied to identify and isolate a tumor-initiating population with stem cell properties in lung cancer.^43^ We therefore performed flow cytometry analysis to examine the existence of SPs in PC9 cells and found that PC9 cells expressing either LSH WT or mutants contain clearly distinct percentages of SPs. Compared with vector control, the SP percentage in PC9 cells stably expressing WT was highly elevated; additionally, PC9 cells expressing R309A have a higher SP percentage than WT, while by contrast, PC9 cells ectopically expressing S503A relative to WT presented a lower SP percentage (Fig. 6f). In accordance with this observation, a flow cytometry assay of cell surface molecule CD338 (also called ABCG2) indicated that WT could increase the expression of ABCG2, which contributes to the SP phenotype by regulating the efflux of Hoechst dye, and R309A induced remarkably elevated ABCG2 expression versus WT; conversely, S503A decreased the expression relative to WT (Fig. 6g). We also obtained consistent results in an Aldefluor assay, which was utilized to determine ALDH activity (Fig. 6h).

It is reported that a variety of H3K4me3-enriched promoters in pluripotent stem cells also contain a repressive histone mark H3K27me3, which could be called as “bivalent histone modifications”.^44^ And there are lots of stem cell-associated genes promoters that are simultaneously marked by both H3K4me3 and H3K27me3 modifications.^45^ Moreover, accumulating evidence suggests that ATP-dependent chromatin remodeling proteins could be implicated in regulating bivalent histone modifications.^46^ Recent research revealed that the distribution of the repressive histone modification mark H3K27me3 is altered obviously in Hells^−/−^ (KO) murine embryonic fibroblasts (MEFs).^47^ Therefore, ChIP assay was applied to explore the possible mechanisms about how LSH promotes stemness-related gene expression. The results demonstrated that the distribution of both H3K27me3 and H3K4me3 marks at the promoters of ABCG2, ALDH1A1 and LGR6 were distinctly changed in LSH WT and mutants. Moreover, the ratio of H3K4me3/H3K27me3 in LSH WT is obviously increased versus Vector, leading to higher expression of these genes in WT. Furthermore, compared with WT, the ratio is as well remarkably elevated in R309A mutant and apparently decreased in S503A mutant (Fig. 6i). These results are consistent with the results obtained from western blot before (Fig. 6d) in our study. All these data indicated that LSH posttranslational modifications may regulate stemness-related genes expression through changing the enrichments of H3K27me3 and H3K4me3 at the promoters of stemness-related genes, such as ABCG2 and ALDH1A1.

**Fig. 7:**
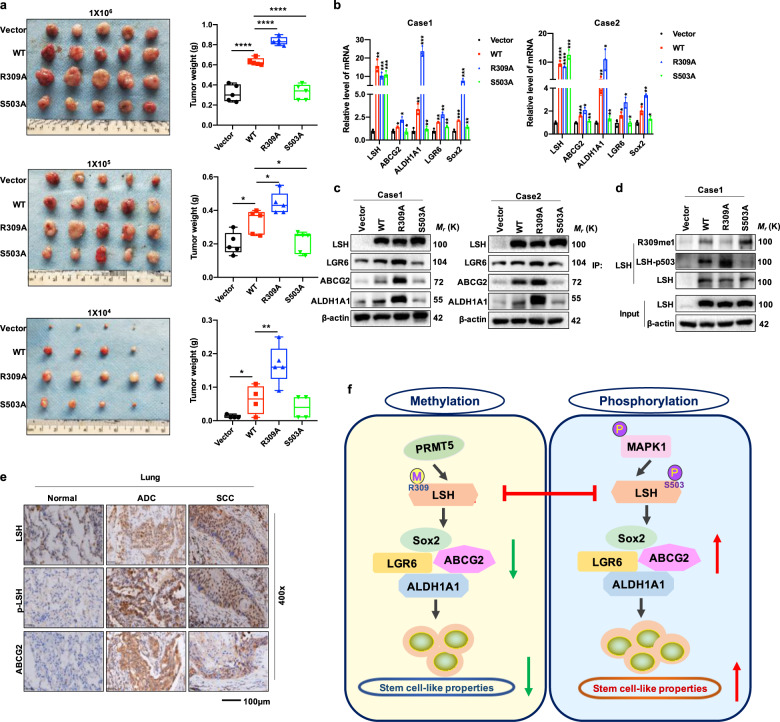
LSH methylation and phosphorylation modulates lung cancer stem-like cells in vivo. **a** An in vivo dilution assay was performed, with representative images of tumors shown (left) and tumor weight recorded (right) (*n* = 5). **b** RT-qPCR analysis in tumors stably expressing Vector, LSH WT, R309A or S503A derived from nude mice was performed to detect mRNA levels of genes associated with stemness. **c** The protein levels of stemness-related genes as indicated were examined by IB analysis in tumors as mentioned in **b**. **d** IP and IB analysis for LSH methylation and phosphorylation with the generated LSH R309me1 and p-S503 antibody in tumors as mentioned in **b**. **e** Representative IHC images of LSH, LSH-p503, and ABCG2 in normal and tumor tissue derived from lung cancer patients. **f** A schematic model to show the posttranslational modifications of LSH and the roles they may play in stemness maintenance in lung cancer: LSH could be methylated at R309 by the protein arginine methyltransferase PRMT5. Meanwhile, LSH could also be phosphorylated by the kinase MAPK1 at S503 residue. Furthermore, LSH S503 phosphorylation could antagonize its R309 methylation, eventually influencing LSH activity to sustain stem cell properties in lung cancer

To further validate whether LSH methylation and phosphorylation could also play crucial roles in cancer in vivo, an in vivo limiting dilution assay was performed using female nude mice to determine the effects of these two PTMs on LSH function in promoting cancer stem cell properties. The nude mice were subcutaneously injected with PC9 cells stably expressing Vector, LSH WT, R309A and S503A at various densities as indicated. Consistent with the results obtained from in vitro experiments, an in vivo study demonstrated that ectopic expression of the LSH R309A mutant increased tumor incidence, size as well as weight relative to LSH WT and Vector control, whereas the LSH S503A mutant exhibited reduced efficiency in terms of tumor formation and decreased tumor size and weight versus LSH WT (Fig. 7a and Supplementary Fig. S6a, b). Consistently, RT-qPCR and IB analysis of tumors from these nude mice showed the same results (Fig. 7b–d). Moreover, we examined the expression of LSH, LSH-p503, and stem cell-associated marker ABCG2 in lung tissue derived from lung cancer patients through immunohistochemistry (IHC) analysis. As expected, the expression of these molecules was significantly higher in tumor tissue than in normal tissue (Fig. 7e), which indicated that there may be clinical relevance among these factors, and further study is needed.

Moreover, there are significantly increased reports indicating that cancer stem cells naturally possess the ability to evade chemotherapy partly because of ABC-transporter expression, and ABCG2 expression has a close correlation with etoposide resistance.^48^ To investigate whether LSH WT and mutants play roles in etoposide resistance, PC9 cells with stable expression of either WT or mutants were treated with etoposide for 48 h. Interestingly, we observed that the R309A mutant, which displayed enhanced stemness characteristics, exhibited a higher capability for etoposide resistance, whereas the S503A mutant, which possessed attenuated stemness properties, was more sensitive to etoposide relative to WT (Supplementary Fig. S7a, b).

Hence, we put forward a notion that LSH could be implicated in regulating lung cancer stemness and chemotherapy resistance. Moreover, there exists a negative correlation between LSH S503 phosphorylation and LSH R309 methylation; LSH S503 phosphorylation may promote stemness properties and chemotherapy resistance, whereas LSH R309 methylation displays the opposite function (Fig. 7f).

## Discussion

ATP-dependent chromatin remodeling enzymes of the SNF2 family, which contains more than twenty subfamilies, possess helicase and ATPase characteristics and can be involved in and play significant roles in a broad range of biological processes, such as chromatin remodeling, methylation, DNA damage repair and gene expression.^49,50^ One of their vital roles is the regulation of stemness and differentiation in diverse cells.^51,52^ Furthermore, increasingly compelling evidence suggests that various alterations of SNF2 family enzymes may participate in the carcinogenesis and development of multiple human cancers.^53^ It is reported that bromodomains (BRDs) of BRG1 and BRM play a key role in stemness maintenance of ESCs (embryonic stem cells) and TSCs (trophoblast stem cells), and these BRDs maybe potentially therapeutic targets for preventing or even eradicating the maintenance of the cancer stem cell phenotype.^54^ Moreover, both BAF45B (DPF1) and BAF45C (DPF3), which belong to the SWI/SNF family, were capable of participating in the regulation of stemness maintenance of glioma initiating cells (GICs).^55^ Additionally, increasing emerging evidence shows that the activities and functions of chromatin remodelers can be modulated by posttranslational modifications of the remodelers themselves. For example, the Ies4 subunit of the INO80 complex could be phosphorylated by the Mec1/Tel1 kinases under DNA-damaging conditions in yeast.^56^ Additionally, as a member of the SWI2/SNF2 superfamily, CBS could be phosphorylated by ATM and CDK2, which may regulate CBS chromatin remodeling activity involved in the DNA double-strand break (DSB) repair pathway.^57^ Furthermore, the acetyltransferase PCAF could acetylate mammalian BRM to negatively impact BRM activities as a chromatin remodeler, including transcriptional activation as well as growth modulation.^58^ However, the methylation of chromatin remodelers alone has been infrequently reported, especially arginine methylation. In our study, we demonstrated that LSH, a member of the SNF2 family, could be phosphorylated at the S503 residue by kinase MAPK1. More importantly and interestingly, LSH can be methylated at the R309 residue by the methyltransferase PRMT5 as well. These two PTMs of LSH can be implicated in manipulating LSH activities to promote the lung cancer stem cell phenotype. Because of its sequence homology to the SNF2 family, we speculate that not only LSH but also some other chromatin remodelers of the SNF2 family may be methylated by PRMT5 and that PRMT5-regulated protein arginine methylation, as one kind of PTM, may also be utilized to modulate chromatin remodeling proteins and then influence their activities and functions.

PRMTs are highly conserved from yeast to human and can be classified into three types, types I, II, and III, which are responsible for catalyzing different types of arginine methylation. So far, eleven PRMTs have been found, of which nine of them are encoded in mammalian genomes, and these PRMTs are capable of participating in multiple cellular pathways including signal transduction, gene transcription and others.^35^ Recently, accumulating evidence indicates a close association between PRMTs and the SWI/SNF family, which may play crucial roles in transcriptional activation or repression. For example, CARM1 (PRMT4), a member of the PRMTs, forms the CARM1-SNF5 complex, which can serve as a mechanism for T3-dependent transcriptional activation.^59^ In addition, CARM1 can methylate chromatin remodeling factor BAF155 at the R1064 residue to facilitate tumorigenesis.^60^

PRMT5 is a type II arginine methyltransferase, which is able to associate with a variety of protein complexes and then methylate histones (H3R8 and H4R3) and nonhistone proteins (E2F1, p53, and others) to induce epigenetic or posttranslational alterations, participating in various critical cellular pathways as well as processes.^61^ Previous studies have suggested that PRMT5 can bind to the SWI/SNF complex to silence some genes through the methylation of arginine residues of histones. For instance, it is reported that PRMT5 is capable of interacting with the ATP-dependent SWI/SNF complex including BRG1 and BRM and methylates H3R8 at the promoter regions of tumor suppressors *ST7* and *NM23*, negatively regulating the expression of these two target genes to take part in modulating cell growth and proliferation.^62^ Furthermore, BRG1 may interact with and recruit PRMT5 to repress Vitamin D-induced Cyp24a1 transcription through H3R8 and H4R3 methylation catalyzed by PRMT5.^63^ Nevertheless, whether PRMT5, an arginine methyltransferase, is able to specifically and differentially methylate ATP-dependent chromatin remodelers is still largely unexplored. In our study, we identified that LSH may serve as a substrate for the arginine methyltransferase PRMT5, and LSH methylation catalyzed by PRMT5 plays significant roles in regulating LSH activity to maintain lung cancer stem cell properties. Moreover, the correlation between LSH and PRMT5 at the mRNA level was analyzed either in The Cancer Genome Atlas (TCGA) database or in lung cancerous tissue, and the results demonstrated that their mRNA levels were positively correlated (Supplementary Fig. S1a). Additionally, we also detected the expression of LSH and PRMT5 at the protein level in lung cancer specimens, identifying that both of them were overexpressed in carcinoma tissue relative to adjacent normal tissue, and their protein levels displayed a positive relationship as well (Supplementary Fig. S1b). Importantly, the KM-PLOT database revealed that lung cancer patients with higher expression of either LSH or PRMT5 may have poorer survival (Supplementary Fig. S1c). These data suggest that LSH have a high correlation with PRMT5. However, it should be pointed out that LSH methylation did not disappear in the R309A mutant (Fig. 2e) and LSH arginine methylation level was obviously reduced but not vanished when PRMT5 was knocked down (Fig. 2k). Therefore, we speculated that R309 seems to be the dominant methylation site, but not the only arginine methylation site of LSH and PRMT5 may not be the exclusive arginine methyltransferase responsible for LSH methylation. Other PRMTs may exist to methylate LSH at other arginine sites, which needs further investigation.

An intriguing finding in our research is that LSH is capable of being fine-tuned both by methyltransferase and phosphokinase. Mounting evidence shows that a molecule can undergo multiple PTMs simultaneously at various sites, among which a complicated connection exists, exerting conjoining influences on protein functions.^64^ Moreover, the interplay between protein methylation and phosphorylation and the roles they may play are receiving increasing attention. Notably, it is reported that EGFR methylation at arginine 1175 positively crosstalks with tyrosine 1173 phosphorylation, which could result in the alteration of EGFR activity.^65^ Recently, a study indicated that PRMT1-mediated PLT3 arginine methylation at the 972/973 sites positively regulated its phosphorylation at the Y696 site.^66^ Similarly, we found that LSH is able to be modified both by methylation at the R309 residue and phosphorylation at the S503 residue; moreover, there exists negative crosstalk between methylation and phosphorylation, which could participate in influencing the activity and functions of LSH in the stemness maintenance of lung cancer. Overall, our study may uncover a novel LSH regulation mechanism at the posttranslational level. Nevertheless, it is as yet unclear whether LSH can be modified by any other PTMs, such as ubiquitination and acetylation, and whether other PTMs crosstalk with LSH methylation or phosphorylation, eventually affecting LSH activity. Hence, there is still much work to be done to thoroughly illuminate LSH PTMs, which may provide a better understanding of its modulation of stemness maintenance in lung cancer.

Previous studies have reported that LSH could be involved in regulating stem cell-like characteristics and is an important modulator during early stem cell differentiation, functioning as a DNA methylation modifier.^67^ More interestingly, LSH is reported as an essential factor that could be implicated in boosting stem-like characteristics and tumorigenesis of skin cells and glioma cells.^22,23^ Nevertheless, the molecular and regulatory mechanisms responsible for modulating LSH activity to regulate stem cell characteristics are still not thoroughly clarified. In this study, we elucidated that LSH was closely involved in lung cancer stem cell maintenance. Moreover, we investigated LSH posttranslational modifications and demonstrated that LSH phosphorylation may promote the expression of stem cell-associated markers, such as ABCG2, ALDH1A1, and SOX2, both at the mRNA and protein levels; meanwhile, LSH methylation may suppress the expression of corresponding genes in lung cancer. Furthermore, the dynamic changes of LSH methylation and phosphorylation may play vital roles in regulating the lung cancer stem cell phenotype. However, the concrete mechanisms by which LSH manipulates the expression of these stemness-related markers are still unexplored, requiring further investigation.

Taken together, we put forward a working model illustrating how a methylation-phosphorylation crosstalk regulates LSH activity and the crucial roles of these two modifications in the stemness maintenance of lung cancer. Although various studies have reported that mutations or epigenetic changes altering the activities and functions of LSH are intimately connected with cancer, the mechanisms underlying these connections remain to be clarified. Further delineation of these molecular mechanisms and pathways will be interesting, challenging and beneficial for enhancing our understanding of LSH functions and developing preventive and therapeutic targets for lung cancer.

## Materials and methods

### Cell culture, plasmids, shRNAs, antibodies, and chemicals

Human 293T cells and the lung cancer line PC9 obtained from the Cancer Research Institute of Central South University were cultured respectively in DMEM (Gibco) and RPMI 1640 (Gibco) medium supplemented with 10% fetal bovine serum (FBS) and 1% penicillin and streptomycin, and all cells were maintained at 37 °C with 5% CO_2_. In addition, all cell lines were subjected to detection of mycoplasma contamination and verified to be negative, and they were passaged <10 times once revived from frozen stocks. Additionally, short tandem repeat profiling was applied to all cell lines to confirm identity.

FLAG-LSH truncation constructs were produced as described before,^20^ the PRMT5 plasmid was purchased from Vigene Biosciences (http://www.vigenebio.com; Shandong, China), and the PRMT5 full-length and deletion mutant constructs were built by subcloning cDNA encoding full or partial open reading frames (ORFs) into the pLVX-EF1α-IRES-Puro vector (catalog no. 631988; Clontech) with an HA tag. Additionally, a prokaryotic expression plasmid of LSH and PRMT5 was generated by inserting their full-length sequences obtained by PCR into the pGEX-4T-1 vector expressing glutathione S-transferase (GST) for expression in *E. coli*. Site-directed mutagenesis of LSH was performed using the QuikChange II Site-Directed Mutagenesis Kit (Agilent) according to the manufacturer’s instructions. The lentiviral shRNA vector against human PRMR5 and nontarget control shRNA were ordered from Genechem (http://www.genechem.com.cn, Shanghai, China). Furthermore, all plasmids were confirmed by DNA sequencing. The sequences of primers for quantitative real-time PCR are listed in Supplementary Table 1.

The following antibodies were obtained from commercial sources and used as primary antibodies: LSH (sc-46665), PRMT5 (sc-376937), MCM3 (sc-365616), RbAp46/48 (sc-373873), HAT1 (sc-390562), and IKKα (sc-7218) were ordered from Santa Cruz Biotechnology. HA-tag (MBL-561) was gained from MBL International Corporation, Flag-tag (TA50011-100) was purchased from Origene, and His-tag (2366), GST-tag (2625), SOX2 (3579), p-IKKα (2697), and Mono-Methyl Arginine (8711) were purchased from Cell Signaling Technology (CST). ABCG2 (MAB46), CXCR4 (AB1846) and β-actin (A5441) were obtained from MilliporeSigma. ALDH1A1 (ab52492) as well as Phosphoserine (ab9332) were obtained from Abcam. Two kinds of secondary antibody, including anti-rabbit (7074) and anti-mouse (7076) IgG HRP-linked antibody, were obtained from CST. In addition, the polyclonal LSH-R309me1 and LSH-pS503 antibodies generated by ABclonal Biotechnology and ChinaPeptides, respectively, were derived from rabbit, and the peptides applied to immunization were LSH 305-315aa (TQEER (me) QKLVRN) and 497-507aa (KETIELS (p) PTGR).

LY3214996 (HY-101494) was obtained from MedChemExpress (MCE), EPZ015666 (S7748), etoposide (S1225) and roscovitine (S1153) were obtained from Selleck, and lambda protein phosphatase (p0735) was purchased from NEB.

### Immunoblotting (IB) and Co-Immunoprecipitation (Co-IP) assay

Immunoblotting (IB) analysis was performed as previously described.^68^ For Co-Immunoprecipitation (Co-IP), cells were harvested and lysed in immunoprecipitation (IP) buffer containing protease inhibitor cocktail (BioTool), and corresponding antibodies were incubated with cell lysates overnight at 4 °C on a rotator; then, Dynabeads^TM^ Protein G (ThermoFisher) was added into the reactions for further incubation at 4 °C for another 2 h. After washing three times with IP buffer, the precipitates resolved with 2**×** SDS sample buffer were subjected to SDS-PAGE and immunoblotted with the indicated antibodies.

### Lentiviral production and infection

To establish stable overexpression cell lines, the lentiviral expression plasmids LSH WT, LSH R309A and LSH S503A were transfected into 293T cells with psPAX2 and pMD2.G (addgene) using transfection reagents according to the directions. The supernatant fractions containing lentivirus were collected 48–72 h after transfection, which were then filtered and used to infect target cells with 5 μg/ml polybrene (Sigma). Cells were screened with 2 μg/ml puromycin for more than 4 days after virus infection. Eventually, immunoblot analysis was performed to verify whether the stable cell lines were successfully generated.

### Peptide synthesis and dot blot analysis

The peptides used for the dot blot assay were synthesized at ABclonal Biotechnology and ChinaPeptides, and the sequences are as follows (the asterisk denotes mono-methylation, while the pound sign indicates phosphorylation):

LSH-R309-WT (aa 305–315): TQEERQKLVRN

LSH-R309me1: TQEE^*^RQKLVRN

LSH-S503-WT (aa 497–507): KETIELSPTGR

LSH-p-S503: KETIEL^#^SPTGR

For the dot blot assay, peptides were diluted with TBS buffer and spotted onto polyvinylidene fluoride (PVDF) membranes, and the membranes were blocked with 5% skimmed milk diluted in TBST buffer after drying at room temperature, followed closely by probing with the indicated antibodies as previously described for immunoblotting analysis.

### In vitro methylation analysis

In vitro methylation analysis was carried out as previously described.^69^ In brief, a prokaryotic expression plasmid pGEX-4T-1-LSH was conducted, GST-LSH fusion protein was expressed in *E. coli* strain BL21 and later purified according to the manufacturer’s directions. HA-PRMT5 fusion protein was obtained from 293T cells transfected with HA-PRMT5 plasmid and later immunoprecipitated with PRMT5 antibody and Dynabeads^TM^ Protein G. GST-LSH fusion protein and immunoprecipitated HA-PRMT5 fusion protein were incubated in reaction buffer containing 1 μM methyl group donor SAM at 30 °C for 60 min. The reaction stopped with SDS sample buffer was subjected to SDS-PAGE and immunoblot with the generated antibody R309me1.

### In vitro kinase assay

In vitro kinase assay was performed as *Bio Protocol* reported before.^70^ GST-LSH fusion protein purified from *E. coli* strain BL21 and His-MAPK1 fusion protein purified from 293 T cells were in incubated in kinase buffer (CST) supplemented with ATP (CST) at 30 °C for 30 min, the reaction terminated with SDS sample buffer was subjected to SDS-PAGE and immunoblot with the generated antibody p-S503.

### Tumor sphere formation assay

Tumor sphere formation analysis was conducted as described before.^71^ In short, cells plated in ultralow attachment six-well plates (Corning), were cultured in serum-free DMEM-F12 medium, supplemented with B-27 supplement (Gibco), 20 ng/ml EGF (Sigma), 20 ng/ml b-FGF (Gibco) as well as N-2 Plus Media Supplement (Gibco). After culturing for 1–2 weeks at 37 °C with 5% CO_2_, cells then formed floating tumor spheres. Eventually, spheres larger than 50 μm in diameter were counted and analyzed. Each sample was repeated at least three times.

### Aldefluor assay, SP analysis and flow cytometry

The Aldefluor assay (STEMCELL Technologies) was performed in accordance with the manufacturer’s instructions and analyzed with FlowJo software.

For SP analysis, cells were labeled with Hoechst 33342 dye (Sigma) according to the instructions reported previously.^43^ Briefly, the cells were harvested, resuspended at 1 × 10^6^ cells/ml in PBS buffer containing 2% FBS, and Hoechst 33342 was added at a final concentration of 5 μg/ml with or without 50 μM Verapamil (Sigma), which was used to inhibit ABC transporters, followed by incubating at 37 °C for 90 min with shaking every 15 min. Then, the cells were washed with ice-cold PBS buffer and centrifuged to collect the cell pellet before resuspending with ice-cold PBS buffer containing 2% FBS. Ultimately, propidium iodide was added to the cell suspension at a final concentration of 1-2 μg/ml (PI, sigma) and used for flow cytometry analysis. The SP cells were selected and sorted by FACS and analyzed with FlowJo software.

### Nude mice and study approval

The xenograft tumor experiment was carried out as previously described.^18^ The 4-week-old female nude mice used in this study were purchased from Hunan SJA Laboratory Animal Co., Ltd. (Changsha, China). The detailed protocol of this experiment is provided in the Supplementary Materials and Methods.

### Statistical analysis

All experiments, except for those involving nude mice, were repeated at least three times. All statistical analysis was performed using Microsoft Excel 2016 and GraphPad Prime version 8.0 for Windows. The unpaired Student’s *t*-test was applied to analyze the differences between two groups, one-way analysis of variance was used to compare multiple groups, whereas Pearson’s correlation coefficient was used for correlation analysis between LSH and PRMT5. The data are presented as the mean ± SD or SEM unless otherwise stated, and *P* values calculated in the corresponding figures are generated from three independent experiments with at least three technical replicates. In these statistical results, ns denotes nonsignificant (*P* > 0.05), * denotes P < 0.05, ** denotes *P* < 0.01, *** indicates *P* < 0.001 and **** denotes *P* < 0.0001.

## Supplementary information

Supplementary Materials
