# Supplementary material for: The cross-talk between methylation and phosphorylation in lymphoid-specific helicase drives cancer stem-like properties
Source: Signal Transduct Target Ther. 2020 Sep 30;5:197. doi: 10.1038/s41392-020-00249-w (PMC7524730; doi:10.1038/s41392-020-00249-w)
Supplement: Supplementary file 1 — Supplementary Materials [file 41392_2020_249_MOESM1_ESM.docx]

Supplementary Materials for

**The cross-talk between methylation and phosphorylation in lymphoid-specific helicase drives cancer stem-like properties**

Na Liu ^1,2,3^, Rui Yang ^1,2^, Ying Shi ^1,2^, Ling Chen ^1,2^, Yating Liu ^1,2^, Zuli Wang ^1,2^, Shouping Liu ^1,2^, Lianlian Ouyang ^4­^, Haiyan Wang ^1,2^, Weiwei Lai ^1,2^, Chao Mao ^1,2^, Min Wang ^1,2^, Yan Cheng­ ^5^, Shuang Liu ^4^, Xiang Wang ^6^, Hu Zhou ^7^, Ya Cao ^1,2^, Desheng Xiao ^1,*^, Yongguang Tao ^1,2,6,*^

*Correspondence to: [taoyong@csu.edu.cn](mailto:taoyong@csu.edu.cn); [xdsh96@21cn.com](mailto:xdsh96@21cn.com),

**This file includes:**

Supplementary Materials and Methods

Supplementary Figures. S1 to S7

Table S1

**Supplementary Materials and Methods**

**Quantitative real-time PCR**

The total RNA was extracted with Trizol (Takara) and reverse transcribed with the PrimeScript™ RT Reagent Kit (Takara) according to the manufacturer’s instructions. The real-time PCR assay was conducted using the Applied Biosystems 7500 Real-Time PCR System with FastStart Universal SYBR Green Master (Roche) and specifically designed primers. The results were normalized to the expression level of ACTB. The 2-^ΔΔCT^ method was utilized to determine the relative quantitation of corresponding gene expression. The sequences of primers for quantitative real-time PCR are listed in **Supplementary Table 1**.

**Cell viability assay**

The cell proliferation assay was performed as described before. In brief, cells (500/100 μl/well) were seeded into 96-well plates after the indicated treatment time with etoposide, 20 μl of 3-(4,5-dimethylthiazol-2-yl)-5-(3-carboxymethoxyphenyl)-2-(4-sulfophenyl)-2H-tetrazolium (MTS, Promega) was added into each well containing cells with 100 μl of culture medium, and the absorbance at 490 nm was determined using a 96-well plate reader (BioTek) to analyze cell viability.

**Immunohistochemistry (IHC) analysis**

IHC analysis was performed as described previously. In short, lung cancer biopsies were validated by a pathologist, Dr. Desheng Xiao (Xiangya Hospital, Central South University, Hunan, China), and obtained from the Department of Pathology in Xiangya Hospital. The slices were incubated with corresponding antibodies, and the images were surveyed and captured using a CX41 microscope (OLYMPUS, Tokyo, Japan), eventually, immunohistochemical scores were evaluated by two pathologists, both from the Xiangya Hospital, Changsha, China.

**GST pull down assay**

The GST pull down assay was performed just as previously reported. In brief, a prokaryotic expression plasmid for LSH with a glutathione S-transferase (GST) tag was constructed, and GST-LSH fusion protein was expressed in *E. coli* strain BL21 and later purified according to the manufacturer’s directions. Purified GST-LSH fusion protein and GST protein were individually incubated with HA-PRMT5 fusion protein derived from 293T cell lysates overnight at 4℃ on a rotator in binding buffer as described before. Glutathione-Sepharose beads were then added into the reactions to further incubate at 4℃ for another 2 h with gentle rotation. After collection and washing three times with binding buffer, the precipitates resolved with 2×SDS sample buffer were subjected to SDS-PAGE and immunoblotted with indicated antibodies.

**Nude mice and study approval**

A xenograft tumor experiment was essentially carried out as previously described*(18)*. Four-week-old female nude mice used in this study were purchased from Hunan SJA Laboratory Animal Co., Ltd. (<http://www.hnsja.com>) and were subcutaneously injected with 1 × 10^6^, 1 × 10^5^ or 1 × 10^4^ PC9 cells stably expressing Vector, LSH, R309A and S503A (5 mice/group). The condition of the mice and the growth of tumors were supervised throughout the whole experiment, with the determination of mouse weight by analytical balance and measurement of tumor size by caliper every other day. The tumors were removed after the mice were sacrificed at the indicated time, and then the volume and weight of tumors were calculated and analyzed.

All experimental procedures for animal study were approved by the Institutional Animal Care and Use Committee of Central South University and strictly complied with the legal mandates and national guidelines for the care and maintenance of laboratory animals. **Supplementary Figures and Figure Legends**

**Figure S1. The correlation between LSH and PRMT5**

(a) The correlation between LSH and PRMT5 at the mRNA level was analyzed either in The Cancer Genome Atlas (TCGA) database or in lung cancerous tissue, including 54 lung ADCs, 60 lung SCCs and corresponding normal lung tissue samples. (b) IB analysis of the expression of LSH and PRMT5 at the protein level in lung cancer specimens and adjacent normal tissue. (c) LSH and PRMT5 expression levels were significantly elevated in lung cancer tissues. Kaplan-Meier curves are shown for the overall survival rates of patients with lung cancer.

**Figure S2. LSH could be methylated at R309**

(a) Mass spectrometric analysis showing LSH R507 and R541 methylation in A549 cells. (b) IB analyses of immunoprecipitated proteins obtained from 293T cells transiently transfected with plasmids expressing LSH WT and LSH mutant (R309/507A/541A). (c and d) Proteins derived from 293T (c) and H358 cells (d) ectopically expressing LSH WT or R309A were subjected to IP analysis, followed by an IB assay with the generated anti-LSH R309me1 antibody. (e) Endogenous arginine methylated proteins were immunoprecipitated from A549 cells treated with EPZ015666 (specifically inhibit PRMT5 methyltransferase activity) by anti-pan mono methyl arginine antibody (Methyl R), and the arginine methylation status of LSH was examined with LSH antibody.

**Figure S3. MAPK1 directly phosphorylated LSH at serine 503**

(a) Various protein kinases potentially responsible for catalyzing LSH phosphorylation at the S503 residue, which were predicted using Group-based Prediction System, are listed in a table. (b and c) After treatment without or with the CDK5 and CDK2 inhibitor roscovitine for the indicated time, 293T cells transiently transfected with LSH were lysed and collected for IP and IB analysis to examined phosphorylated LSH. (d) IP and IB analysis of the phosphorylated LSH in 293T cells after treatment without or with IKKα inhibitor for the indicated time. (e) After treatment with the MAPK1/2 inhibitor LY3214996 for the indicated time, an IB assay was carried out to detect the phosphorylated LSH with an anti-phosphoserine antibody. (f) IB analysis was used to detect MAPK1 and p-MAPK1 in H1299 cell lines, which MAPK1 was stably knocked down using MAPK1 short hairpin RNAs (shRNA).

**Figure S4. MAPK1-mediated LSH phosphorylation crosstalk with methylation of LSH by PRMT5**

(a) IP and IB analysis for LSH methylation with the Methyl R antibody in 293T cells transiently transfected with LSH WT and mutants, including R309A as well as S503A. (b) IP and IB analysis for LSH methylation with the generated LSH R309me1 antibody in PC9 cells stably expressing LSH, R309A and S503A.

**Figure S5. LSH methylation and phosphorylation modulate the lung cancer stem cell phenotype in vitro**

(a) Gene Ontology (GO) enrichment analysis of RNA-seq data was conducted using DAVID tools, and the visualized results of biological process (BP) are shown in a bubble diagram, in which the stem cell-associated BPs are marked in bold. (b) RT-qPCR analysis in PC9 cells stably expressing Vector, LSH WT, R309A or S503A was performed to detect mRNA levels of genes associated with stemness. (c) IB analysis was used to detect LSH in the cytoplasm and nuclear protein from PC9 cells stably expressing Vector, LSH WT, R309A or S503A. (d) RT-qPCR analysis in PC9 cells stably expressing Vector, LSH WT, R309A or S503A was used to detect mRNA levels of FADS2 and SCD1.

**Figure S6. LSH methylation and phosphorylation modulate the lung cancer stem cell phenotype i****n vivo**

(a and b) An in vivo limiting dilution assay was carried out, and mouse status as well as weight were monitored at the indicated time points. Nude mouse body weight was (a) shown, and tumor volume (b) was calculated according to the following formula: tumor volume (mm^3^) = longer diameter × shorter diameter^2^ × 1/2.

**Figure S7. LSH methylation and phosphorylation modulate the drug resistance of lung cancer cells**

(a and b) Drug resistances of PC9 (a) and H358 cells (b) stably expressing Vector, LSH WT, R309A or S503A. Cells were exposed to etoposide for 48 hours, and then cell viability was measured by MTT assays.

**Table S1.** **Sequences for RT-PCR primers**

| **Name** | **Gene ID** | **Primer** |
| --- | --- | --- |
| ABCG2 | NM_004827 | F: GTTCTCAGCAGCTCTTCGGCTT |
|  |  | R: TCCTCCAGACACACCACGGATA |
| ALDH1A1 | NM_000689 | F: CGGGAAAAGCAATCTGAAGAGGG |
|  |  | R: GATGCGGCTATACAACACTGGC |
| c-Myc | NM_002467 | F: CCTGGTGCTCCATGAGGAGAC |
|  |  | R: CAGACTCTGACCTTTTGCCAGG |
| OCT4 | NM_002701 | F: CCTGAAGCAGAAGAGGATCACC |
|  |  | R: AAAGCGGCAGATGGTCGTTTGG |
| Nanog | NM_024865 | F: CTCCAACATCCTGAACCTCAGC |
|  |  | R: CGTCACACCATTGCTATTCTTCG |
| LGR5 | NM_003667 | F: CCTGCTTGACTTTGAGGAAGACC |
|  |  | R: CCAGCCATCAAGCAGGTGTTCA |
| LGR6 | NM_021636 | F: GAGATGGAGGACTCAAAGCCAC |
|  |  | R: AGTCCATTGCAGAGCACGGAGA |
| Sox2 | NM_003106 | F: GCTACAGCATGATGCAGGACCA |
|  |  | R: CGAGTAGGACATGCTGTAGGT |
| KLF4 | NM_004235 | F: CATCTCAAGGCACACCTGCGAA |
|  |  | R: TCGGTCGCATTTTTGGCACTGG |
| CD44 | NM_000610 | F: CCAGAAGGAACAGTGGTTTGGC |
|  |  | R: ACTGTCCTCTGGGCTTGGTGTT |
| CD24 | NM_013230 | F: CACGCAGATTTATTCCAGTGAAAC |
|  |  | R: GACCACGAAGAGACTGGCTGTT |
| CXCR4 | NM_003467 | F: CTCCTCTTTGTCATCACGCTTCC |
|  |  | R: GGATGAGGACACTGCTGTAGAG |
